# A Framework of a Health System Responsiveness Assessment Information System for Iran

**DOI:** 10.5812/ircmj.17820

**Published:** 2014-06-05

**Authors:** Somayeh Fazaeli, Maryam Ahmadi, Arash Rashidian, Farahnaz Sadoughi

**Affiliations:** 1 Department of Health Information Management, School of Health Management and Information Sciences, Iran University of Medical Sciences, Tehran, IR Iran; 2 Department of Health Management and Economics, School of Public Health, Tehran University of Medical Sciences, Tehran, IR Iran; 3 Knowledge Utilization Research Center, Tehran University of Medical Sciences, Tehran, IR Iran

**Keywords:** Health, Information System, Iran

## Abstract

**Background:**

Responsiveness assessment of health system with the quality information is the key in effective evidence-based management of the health system.

**Objectives:**

This qualitative study defines the necessary components required for the health system responsiveness assessment information system (HS-RAIS).

**Materials and Methods:**

This study was conducted based on mixed-methods approach and by using Delphi technique (29 participants in first round and 25 participants in second round) and semi-structured interviews in Iran 2013. The participant selection strikes a balance between being able to provide valid data, and increasing representative’s leverage. The final framework for HS-RAIS was extracted from in-depth interviews with ten key informants.

**Results:**

We followed these recommendations and developed a framework in 10 components including: minimum datasets, data sources, data gathering, data analysis, feedback and dissemination, legislative needs, objectives of health system responsiveness assessment, repetition period, executive committee and stewardship.

**Conclusions:**

This framework provides useful information for decision-making at all levels about assessment of health system.

## 1. Background

The world health report 2000 introduced a new framework for the assessment of health system performance. Within this framework, health systems responsiveness to people’s non-medical expectations was identified as a key goal to each health systems contribute in addition to improving population health and fairness of financing (1). One of the most perfect tool for responsiveness assessment is the WHO’s responsiveness index (2). Responsiveness indicator is a weighted composite index, including eight dimensions. Each dimension is in turn covered by several items of questionnaire in responsiveness module. These dimensions are dignity, autonomy, confidentiality of information, communication, prompt attention, quality of basic amenities, access to support, and choice (of health care provider) which are classified into two general categories, including respect for persons and client orientation (3). Both the level and the distribution in health system responsiveness are measured (3). In May 2001, WHO held a planning meeting to gather the experts’ views on the concepts and methods for measuring the responsiveness of health system. The participants agreed on the importance of using representative samples, developing a short questionnaire in capable of being used alongside other surveys in countries, and the need for a comprehensive data collection strategy to assess responsiveness. It was recommended that the countries required training materials and resources explaining how to carry out the surveys effectively. In addition, linking to policy through the involvement of stakeholders in the process of responsiveness assessment, developing appropriate “reporting systems” to share findings with facilities and consumers and studying the effect of legislation related to responsiveness issues were recommended (3). To meet these recommendations, it is important to evaluate the current opportunities in each country and design comprehensive approaches toward responsiveness assessment. We have approached the issue from the information system perspective. Information system has been defined as an arrangement of information (data), processes, people, and related technology that interact to collect, process, store and provide as output the information needed to support the organization (4). In Iran, several registration systems have been developed for communicable and non-communicable diseases and preventive measures (5, 6). But there are no integrated health information systems that meet the requirement for the health system, and respond to the requirements of assessing responsiveness. Hence, in spite of the importance of the issue and the efforts of health system responsiveness assessment, the current information system is far from optimum (7).

## 2. Objectives

The present study has been conducted to design the information system for assessing health system responsiveness based on the WHO recommendations and national experiences, resources and needs.

## 3. Materials and Methods

In this mixed method (combined of comparative and qualitative) study, we conducted a comprehensive review of literature and guidelines about national health information systems components and health system responsiveness assessment to develop a preliminary framework for the information system, which we called the "Health System Responsiveness Assessment Information System (HS-RAIS)". The available published literature was searched on the Web of Science, Pubmed, Ovid, Science Direct, Google Scholar, WHO website, SID, Iranmedex and Magiran with keywords such as: responsiveness, responsiveness assessment information system, health information system, national health information system, responsiveness assessment. Reference lists of the retrieved articles were also followed. We excluded literature related to other systems mentioned above. Documents up to the end of 2012 were included. We identified 58 documents (31 studies related to responsiveness assessment, 23 documents about health information system and four documents about Iranian national survey) with relevant scopes. This study was employed an experts’ panel (including the authors of the current study) to screen identified documents based on providing a relatively comprehensive coverage of issues relevant to HS-RAIS. We screened identified documents (Based on an expert’s panel including the authors of the current study) and selected 18 studies provided a relatively comprehensive coverage of issues relevant to HS-RAIS. Finally, nine documents were selected related to responsiveness assessment (1, 3, 8-13), seven documents about health information system (5, 6, 14-18) and two documents about Iranian national survey (20, 21). We found three documents related to responsiveness assessment in Iran (that one study was selected after the screening (13) and six documents about health information system with relevant scopes (that selected three studies (5, 6, 14)). In our literature review, there was no document completely related to the HS-RAIS. In the next step, to provide a preliminary HS-RAIS framework for Iran, we conducted an expert panel based on the findings of the literature review. The data was collected through interviews (Semi-structured interview) between August and September 2012. Interviews approximately lasted about 45 to 80 minutes until saturation of concepts was reached. In this step, all possible options for components in the preliminary framework were considered. Components of the preliminary framework of HS-RAIS were validated by the Delphi technique.

### 3.1. Panel Members and Setting

Purposive sampling through expert sampling was initially used for data collection. To ensure the representativeness of the panel members, they were selected from the three groups consisting of: faculty members in related fields, related informant managers and policy makers in health system of Iran and health services providers who have at least five years of relevant experience. Overall, the panel member selection strikes a balance between being able to provide valid data, and increasing representative’s leverage (Table 1).

**Table 1.** Demographic Characteristics of Participants in the First and Second Round of Delphi^a^

Demographic Characteristics of Participants	First Round	Second Round
**Gender**		
Female	5 (17)	4 (16)
Male	24 (83)	21 (84)
**Level of Education**		
PhD	24 (83)	20 (80)
PhD Student	4 (14)	4 (16)
Masters	0 (0)	0 (0)
Bachelor	1 (3)	1 (4)
**Discipline**		
Medical	6 (21)	4 (16)
Nurse	1 (3)	1 (4)
Health information management and health care management	10 (34)	9 (36)
Other disciplines	12 (41)	11 (44)
**Work Experience**		
Less than 10 years	13 (45)	10 (40)
10 to 20 years	9 (31)	9 (36)
More than 20 years	7 (24)	6 (24)

^a^Data are presented as No. (%).

A snowball sampling strategy was used to identifying participants. Snowball sampling begins by identifying participants through direct contacts and asking each participant to recruit others. It helped us to reach Individuals who were not in the initial list. The panel members was comprised of eight key informants to provide a preliminary HS-RAIS framework, 35 individuals in Delphi stage (29 and 25 responded in the first and second round respectively) and ten key informant in final stage. Participants (six in first and four in second round of Delphi) did not participate due to their busy work life. The interviews to access the comments of participants in the Delphi were conducted in email and face-to-face approach.

### 3.2. Consensus Development

In the first round we sent questionnaires to the panel members consist of components of Preliminary framework for HS-RAIS, the scoring system and the definitions. Were asked panel members to give their opinions about each component in three options: agree (scored: 1), disagree (scored: -1) and without opinion (scored: 0). Components with more than 75 percent Consensus were used for the secondary framework. Components with agreement between 50 to 75 percent were entered into the second round of Delphi and items that acquired less than 50 percent of total agreement were excluded. After gathering the panel member’s comments, we summarized the opinions in a suitable format for feedback, so that each member received a summary of the panel opinions as well as a reminder of the scores that the member had assigned to each component. Then the panel members were invited to a second round. Based on the results of data analysis in second round of Delphi, remained no components with agreement between 50 to 75 percent. In the final stage of consensus development, the panel members were invited (face-to-face panel meeting) to view the feedbacks, and review and discuss their opinions about appropriateness of each category and components based on their own professional judgment. The interviews focused on the applicability, adaptation, relationship between components and approaches to improve data collection and future steps in development of HS-RAIS for Iran. All interviews were conducted after setting the time, explaining the aim and the processes of the study. Consent was taken from all participants. In this stage, the interviews were semi-structured and a guided questionnaire was employed to guide the discussion. Interviews approximately take about 50 to 120 minutes, until saturation of concepts was reached. All interviews were audio recorded and transcribed in a convenient time. Two of the authors and three of participants participated in data coding process. To avoid bias in the interviews, all interviews were conducted by the principal investigator [S.F.]. The interviewer was a PhD student in the field of health information management and had 10 experiences in qualitative studies. To avoid potential bias caused by the presence of the principal investigator in all phases of the study, various stages of this study were controlled by two experts. The sample size was determined according to achieve a level of saturation. Delphi and final stage of the study was done between September 2012 and April 2013.

## 4. Results

Based on the review of literature on responsiveness assessment, information system components and Iran’s national surveys, a preliminary framework was developed for HS-RAIS including three main components: inputs (divided in six categories and 37 subcategories), processes (divided in three categories and 16 subcategories) and outputs (divided in two categories and eight subcategories). As shown in Table 2, 31 sub-categories in the first round of Delphi gained score higher than 75 percent of total points and seven items (with agreement between 50 to 75 percent) were entered into the second round of Delphi and 23 sub-categories that scored less than 50% of total points were excluded. The panel members added a further 4 sub-categories to the list in the second Delphi round, that scored less than 50% of total points and were excluded (Table 2).

**Table 2.** Main Components of Health System Responsiveness Assessment Information System

Categories	Delphi Score	
	Round No. 1	Round No. 2
**Inputs**		
**Required datasets^a^**		
Household questionnaire		
Household care	83	-
Health insurance	100	-
Permanent income indicators	86	-
Household expenditure	91	-
Individual questionnaire		
Socio demographic characteristics	93	-
Health state descriptions and valuations	76	-
Risk factors	79	-
Coverage	79	-
Health goals and social capital	58	77
**Objective of health system responsiveness assessement** ^**b**^		
Assessment of interventions to improve the health system responsiveness	93	-
Compare responsiveness in different health Insurance organizations	76	-
Compare responsiveness between the public and Private sector	79	-
Compare responsiveness in the outpatient and inpatient services	79	-
Indicate responsiveness of family physicians at different regions in country	58	80
Assess adaptation with standards of health system performance	93	-
**Stewardship (Oversight)** ^**c**^		
The Joint committee (with representatives of provider, consumer and purchaser of health services)	62	84
Executive committee^c^		
The Joint committee (with representatives of provider, consumer and purchaser of health services)	62	80
**Staff and their capabilities** ^**d**^		
Health information management	86	-
Health care management or health policy	90	-
Health economic	76	-
Statistics	83	-
**Legislative Needs**		
Necessity of legislation by the legislative authorities to implementation of health system assessment	86	-
Necessity of confidentiality of personally identifiable information of Participants in health system responsiveness assessment	86	-
Necessity of signed an informed consent for participants in health system responsiveness assessment	79	-
Necessity of legislation for the various stages of the assessment process (including abuse and neglect, and)	93	-
**Process**		
**Sampling** ^**e**^		-
Multistage sampling (provincial and urban-rural)	79	-
**Data Sources And Data Gathering Methods** ^**f**^		-
Population-based survey :face to face interview	83	-
Patients :telephone/Face to face interview	62	76
Healthcare professionals: telephone/face to face interview/Email	66	80
**Repetition Period of Assessment** ^**g**^		-
Every five years	52	76
**Outputs**		
**Users and information feedback tools**		
Reporting based on the standard forms on the Website of Stewardship ministry	90	-
Reporting based on the standard forms to policymakers of the ministry of health and Related units in universities	79	-
Reporting based on the standard forms to providers	76	-
Reporting based on the standard forms to population	79	-
Publishing article in journals and magazines	83	-
**Media for information feedback**	90	-
website	86	-
Email	77	-
Journals and magazines	76	-

^a^In this category health occupations and mortality were excluded.

^b^In this category accreditation and ranking of the health system institutions and assessing employee performance of the health system were excluded.

^c^In this category ministry of Health, ministry of labor and social affair, statistical center, health Insurance organization, medical council (as a nonprofit NGO) and private sector along with vice-presidency for strategic planning and supervision and non-governmental opinion poll agencies as new suggested sub-categories in second round of Delphi were excluded.

^d^In this category, there was no general agreement for the presence of computer, physician, nurse, paramedical and health insurance specialists. Epidemiologist and social sciences specialist as new suggested sub-categories in second round of Delphi were excluded.

^e^In this category regional (based on the human development index), provincial, urban-rural, and multistage sampling (regional and urban-rural) were excluded.

^f^In this category population-based survey-telephone, population-based survey-postal/self-administered and population-based survey-combined approaches (face to face interview-telephone-postal/self-administered) were excluded.

^g^In this category annual, every two years, every three years and more than every five years were excluded.

**Figure 1.**
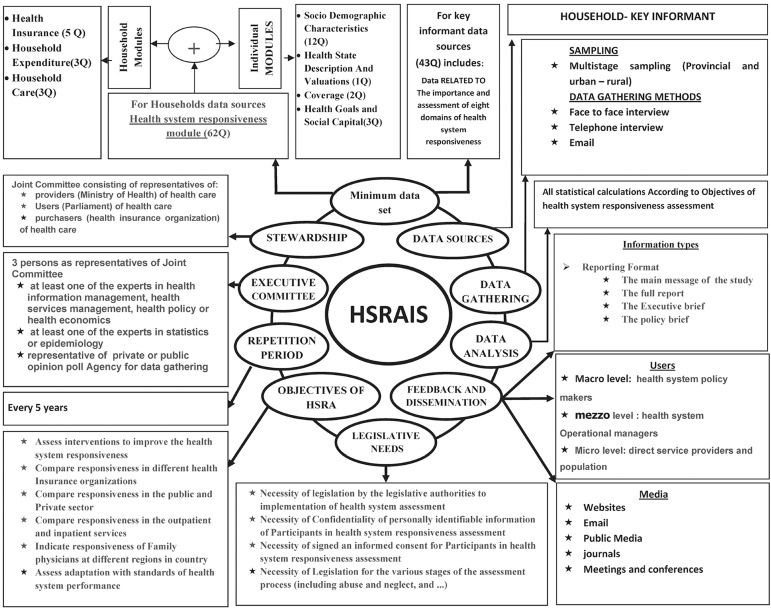
The Suggested Framework for a Health System Responsiveness Assessment Information System (HS-RAIS) for Iran

Secondary framework (obtained from two rounds of Delphi technique) was examined by 10 informants. Interviews with the key informant demonstrated the new dimensions of HS-RAIS. All participants agreed on the importance of joint committee as a stewardship of health system responsiveness assessment and more use of information and communication technologies (ICT) such as email in data collection was recommended. The participants suggested three parts for feedback and dissemination category including: Information types, users and media. The components of each part were extracted from the components of users and information feedback tools and media for information feedback category (Table 2). All participants agreed on the household as a best option for data sources to measuring responsiveness. The participants suggested the consideration of key informant surveys as a complementary measure of health system responsiveness. In this category there were no agreements on the use of patient as a data source. Finally, according to Table 2, participants proposed the new structure for executive committee. In this regard, specialists were categorized into two homogeneous groups and the participants suggested that at least one person must be present in the committee from each groups. Our final framework for an integrated HS-RAIS is shown in Figure 1.

## 5. Discussion

Responsiveness to people’s non-medical expectations is now seen as a key characteristic of effective health systems. In this regard, policy-makers and providers of health services should consider how to narrow the gap between public expectations and experiences of health services recipients (19, 20). Responsiveness assessment of health system with the quality information is the key to effective evidence-based management of the health system (21). But in Iran, lack of an integrated health information system for responsiveness assessment was a stumbling block on the way to assessment of health system goals (7). The designed framework in this study seems to be integrated and appropriate framework to assessment of health system responsiveness in Iran. According to the findings, this final framework is including 10 components: minimum datasets, data sources, data gathering, data analysis, feedback and dissemination of information, legislative needs, objectives of health system responsiveness assessment, repetition period, executive committee and stewardship. HS-RAIS has more components compared to WHO framework for health information system which is divided into six components (15). Both HS-RAIS and WHO framework for health information system components are grouped under three headings: inputs, process and outputs. According to the technical consultation on stewardship in health systems-that was organized by WHO the participants produced a list of possible stewardship tasks, most of which fit into the three part classification: formulating health policy, exerting influence and collecting and using intelligence (3). In our HS-RAIS framework, stewardship is exerted via a joint committee consisting of representatives of providers (ministry of health and medical education), users’ representatives, and purchasers (e.g. health insurance organization) of health care services. The designed HS-RAIS has predicted the specialty of executive committee members and legislative needs for health system responsiveness assessment. Zolala (2011) has noted in her study that introducing appropriate rules and allocating sufficient resources, including human resources and appropriate staff training as suggestions to strengthen health information systems in Iran (14). There are three main types of data sources currently being used or tested by WHO, to measure responsiveness. They are: facility-based (this consists of four components: management, staff, patient), population-based and key informant surveys (while only reflects “expert opinion”). In our study a wide number of useful suggestions were received about the strengths of population-based and key informant surveys than facility-based ones (especially patients) as the data sources to the responsiveness assessment (3, 11). Participants argued that respectively use of households and key informant surveys was more useful to satisfy the HS-RAIS objectives. Considering the feedback modes, we suggest that information about responsiveness assessment should be provided for three levels of users: Macro level including health system policy makers, meso level including health system operational managers (e.g. district health centers, teaching and district general hospital, medical universities, relevant research institutes, newspapers and broadcasters) and micro level including direct service providers and users (e.g. doctors, nurses, researchers, reporters and people). These users can be divided into internal and external users (5). The main strong point of this study was to focus on the framework for performance assessment of health system. The most important limitation of this study was the use of experts living in Iran lonely and for this; we have limits to generalization of our results for other countries. In addition some limitations should be considered in interpreting our findings. We tried to conduct a comprehensive review of the literature, but our review was limited to available documents on the internet (not for documents about Iran). Therefore, our findings regarding health information system and responsiveness assessment in the other countries may not be comprehensive. In addition, further studies are necessary to evaluate our framework in terms of its effectiveness and efficiency. Lack of accurate data on responsiveness is a major barrier in the health policy-making process for health system responsiveness assessment in Iran (12). Activities and institutions are still in their infancy and most of the requirements for HS-RAIS have not been designed or implemented. Therefore, no formal information systems or reporting systems for health system responsiveness assessment have been implemented in Iran (12). Therefore, an appropriate framework for assessment of health system responsiveness is necessary. The framework presented in this study covers different dimensions and all stages of health system responsiveness assessment process. This framework can also be suitable for other countries in the Eastern Mediterranean Region and other similar countries.

### 5.1. Ethical Considerations

Ethical issues (Including plagiarism, Informed Consent, misconduct, data fabrication and/or falsification, double publication and/or submission, redundancy, etc) have been completely observed by the authors.
